# Repetitive DNA landscape in essential A and supernumerary B chromosomes of *Festuca pratensis* Huds

**DOI:** 10.1038/s41598-019-56383-1

**Published:** 2019-12-27

**Authors:** Rahman Ebrahimzadegan, Andreas Houben, Ghader Mirzaghaderi

**Affiliations:** 1 Department of Agronomy and Plant Breeding, Faculty of Agriculture, University of Kurdistan, 6617715175 Sanandaj, Iran; 2 Leibniz-Institut für Pflanzengenetik und Kulturpflanzenforschung (IPK) Gatersleben, Corrensstrasse 3, 06466 Stadt Seeland, Germany

**Keywords:** Plant genetics, Genetics, Cytogenetics

## Abstract

Here, we characterized the basic properties of repetitive sequences in essential A and supernumerary B chromosomes of *Festuca pratensis* Huds. This was performed by comparative analysis of low-pass Illumina sequence reads of B chromosome lacking (−B) and B chromosome containing (+B) individuals of *F. pratensis*. 61% of the nuclear genome is composed of repetitive sequences. 43.1% of the genome are transposons of which DNA transposons and retrotransposons made up 2.3% and 40.8%, respectively. LTR retrotransposons are the most abundant mobile elements and contribute to 40.7% of the genome and divided into Ty3-gypsy and Ty1-copia super families with 32.97% and 7.78% of the genome, respectively. Eighteen different satellite repeats were identified making up 3.9% of the genome. Five satellite repeats were used as cytological markers for chromosome identification and genome analysis in the genus *Festuca*. Four satellite repeats were identified on B chromosomes among which Fp-Sat48 and Fp-Sat253 were specific to the B chromosome of *F. pratensis*.

## Introduction

The genus *Festuca* consists of more than 450 species^1^ and is one of the largest genera in the Poaceae. *Festuca* sp. and its closely allied genus *Lolium* sp. belong to the grass family Poaceae, subfamily Pooideae and tribe Poeae^2^. The genus *Festuca* is an ancient group and considered as one of the main evolutionary lines in the tribe Poeae^3^. *Festuca* species have diverse distribution and are considered as important components of grass ecosystems of the temperate zones^4^. Ten to 12 species of *Festuca* grow wildly throughout Iran^5,6^.

Species inside *Festuca* vary substantially in DNA C-value and ploidy levels, from diploid (2*n* = 2*x* = 14) to dodecaploid (2*n* = 12*x* = 84), most of them being allopolyploid^7–9^. There are many cytogenetic studies of different populations of the genus *Festuca*^7,10,11^.

*Festuca pratensis* Huds. (Meadow fescue) is a diploid species in the genus *Festuca* with 2*n* = 2*x* = 14 standard (A) chromosomes (Fp genome). In addition to the essential A chromosomes, one to five supernumerary B chromosomes (Bs) have been reported in *F. pratensis*, however one and two Bs are common in B-carrying individuals^12–14^. Bs are generally neutral and may have negative effects on the host when they are present in higher numbers. In some plants such as *F. pratensis*, *Secale cereal* and *Allium schoenoprasum*, Bs may confer adaptive advantage under the unfavorable conditions^15–17^. There are reports suggesting that Bs increase allelic variation by altering the frequency and distribution of crossing overs in A chromosome^18–20^ suggesting that Bs are not genetically inert and directly or indirectly affect regulatory mechanisms in the cell.

Transposons and satellites (satDNA) are two major groups of repetitive sequence in eukaryotic genomes and have diverse sequence and distribution patterns^21–25^. The length of satellite monomers can vary widely but are typically a few hundred nucleotides^26,27^. Commonly, satellite repeats form tens of kilobases to megabases structural blocks with biased base composition^28^. The rapid evolution of satellite repeats in plant genomes, has led to their intraspecific homogenization and fixation of species-specific polymorphisms, making satDNA families unique source of molecular and cytogenetic markers to analyze genetic diversity and genome evolution^28–32^. Repetitive sequences also have accumulated in B chromosomes during the process of B chromosome formation and evolution^33^.

With the availability of high throughput sequencing technologies and development of assembly-free methods for assessing repetitive sequences^34^, we were interested to conduct a genome wide repeatome analysis in *F. pratensis*. Its 1C DNA value is about 3.25 pg (≈3.178 Gbp) as measured for the cultivar ‘Kolumbus’^35^. Physical mapping of ribosomal DNA sites in some *Festuca* species revealed that 5S and 45S ribosomal loci in *F. pratensis* have been localized on the short arms of chromosomes 2 and 3, respectively^36^. FISH using BAC clones^37^ and sequencing of chromosome 4F^38^ have identified some repetitive DNA sequences in the meadow fescue genome. Using five tandem repeats identified from chromosome 4F together with rDNA probes, Křivánková, *et al*.^39^ established a molecular karyotype of meadow fescue. Majka, *et al*.^40^ identified an A chromosome-specific repetitive sequence in this species.

In order to characterize the repeatome landscape of *F. pratensis* in plants with and without B chromosomes, we employed low coverage next generation sequencing in combination with the RepeatExplorer software tool and FISH to determine the types, abundance, organization and chromosomal position of repetitive DNA sequences. The first B-specific tandem repeats of *F. pratensis* decorating entire B chromosomes after FISH were identified.

## Results

### Identification and classification of transposable elements

In total, 17 million Illumina 150 bp paired end reads were generated from both −B and +2B *F. pratensis* genotypes corresponding to about 0.4× coverage of the haploid genome. The GC content for the genome showed a value of 46%. Comparative analysis of reads from −B (250410 reads) and +2B (249590) genotypes by the RepeatExplorer pipeline^41^, showed that highly and moderately repetitive sequences constitute 61% of the nuclear genome. The overall proportions of individual repeat types and monomer frequencies (except for B enriched repeats) was similar in the −B and +2B genotypes (Supplementary Table S1).

**Figure 1:**
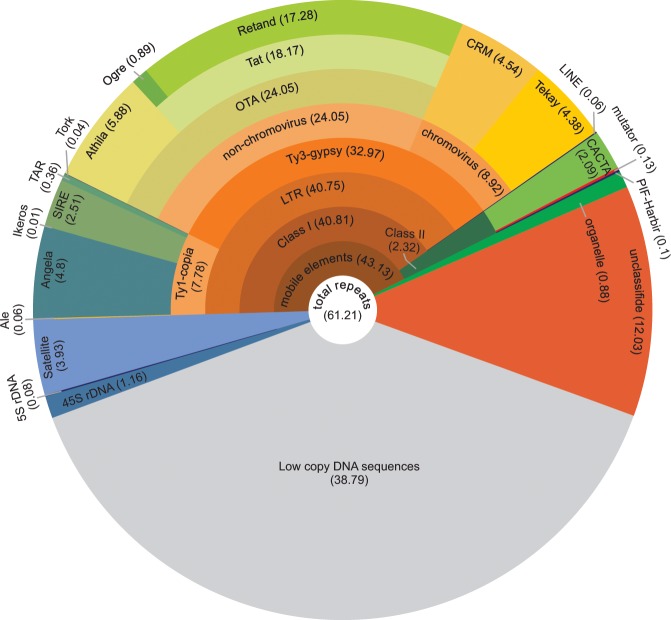
Types of highly and moderately repeated DNA sequences in *F. pratensis* genome. The proportion of each repeat type or family is shown inside parenthesis. Repeat proportions were calculated by annotating clusters representing at least 0.01% of the genome in RepeatExplorer. For mobile elements, members of each repeat family is indicated in outer layers.

43.13% of the repeats are composed of transposons with majority (40.81%) of which being retrotransposons. On the other hand, class II transposons contributed to only 2.32% of the repeats. LTR retrotransposons are the most abundant mobile elements and composed 40.75% of the genome. LTRs divided to Ty3-gypsy and Ty1-copia super families with 32.97% and 7.78% of genome, respectively. More details about the types and proportions of the identified repeats are presented in Fig. 1.

### Satellite repeat identification

Using the tandem repeat analyzer (TAREAN) pipeline^42^, eighteen different satellite repeats representing 3.93% of the genome in the *F. pratensis* were *in silico* identified (Table 1). Five clusters were identified as high confident putative satellites and thirteen clusters as low confident putative satellites. The proportion of each of these tandem repeats in the genome and other details including consensus length and satellite probability are shown in Table 1. All these repeats exhibited graphics with a globular or circular layout and showed C (Connected component index) and P (Pair completeness index) indices close to 1. Both C and P are close to 1 for putative satellite repeats. Although the parameter C facilitates the identification of clusters representing tandemly repeated genomic sequences by being close to 1, it does not efficiently discriminate clusters derived from satellite DNA from those representing other types of tandem repeats. In fact, the proportions of broken pairs are much higher in tandem repeats scattered in the genome in a high number of short arrays^42^. Fraction of complete pairs in cluster is assessed by the pair completeness index P^42^.

**Table 1:** Characterization of satellite DNAs identified in the genome of *F. pratensis*.

	*Fp*-Sat	Genome Proportion [%]	Size*real	Satellite probability	Consensus length	Connected component index C**	Pair completeness index P***	Blast homology search result
High confidence	2	1.1	5573	0.741	317	0.952	0.887	82% identity with repetitive sequence of *L. perenne* (AF063226.1)
	48	0.49	2451	0.908	667	0.96	0.918	—
	131	0.12	608	0.748	659	0.964	0.848	—
	162	0.05	250	0.815	346	0.944	0.908	96% identity with *F. pratensis* TR15 seq. (JX624136.1)^38^
	253	0.015	77	0.948	372	0.987	0.925	—
Low confidence	37	0.56	2817	0.679	347	0.923	0.927	—
	71	0.36	1795	0.0184	956	0.719	0.681	—
	75	0.33	1633	0.022	136	0.734	0.825	—
	84	0.3	1513	0.531	343	0.929	0.891	98% identity with *F. pratensis* satellite TR7 seq. (JX624133.1)^38^
	124	0.14	707	0.612	173	0.928	0.916	96% identity with *F. pratensis* satellite TR5 seq. (JX624131.1)^38^
	130	0.12	623	0.0344	305	0.788	0.653	—
	133	0.12	605	0.0746	175	0.86	0.885	100% identity with *F. pratensis* satellite TR6 seq. (JX624132.1)^38^
	159	0.052	259	0.057	340	0.869	0.786	—
	161	0.05	252	0.0952	660	0.905	0.8	80% identity with *Avena strigosa* beta-amyrin synthase (Sad1) and cytochrome P450 CYP51H10 (Sad2) genes, complete cds (DQ680849.1)^65^
	175	0.035	176	0.0424	842	0.795	0.725	—
	203	0.024	122	0.0318	208	0.852	0.649	94% identity with *F. pratensis* satellite TR11 seq. (JX624134.1)^38^
	219	0.02	98	0.582	383	0.898	0.96	97% identity with *F. pratensis* satellite TR12 seq. (JX624135.1)^38^
	236	0.017	84	0.159	125	0.905	0.867	—

*Number of reads in the cluster.

**Proportion of nodes of the graph which are part of the largest strongly connected component.

***Proportion of reads with available mate-pair within the same cluster.

### Physical mapping of satellite repeats

The consensus monomers of five tandem repeats were selected for PCR and FISH analysis based on their frequency in −B and +B genotypes and satellite probability (Table 2, Supplementary Table S1).

**Table 2:** Repeats used in FISH experiments including those significantly enriched on B chromosomes or produced banding patterns on A chromosomes of *F. pratensis*.

Repeat	Cluster	Repeat type	Monomer bp	Reads		Genome %		2B*100/(0B + 2B)	FISH signal on B	PCR Primers for amplification of satellites (5′ ->3′)
				−B	+2B	−B	+2B			
Fp-Sat2	2	Tandem	317	2713	2860	0.53	0.56	51	−	F: ACC CCC TAA CCC TAA ACT CTG T R: AGC CTG GAT CCT TCT TAG TCA C
Fp-Sat37	37	Tandem	347	1193	1624	0.25	0.34	57	+	F: CAT GCC TAA TTG CCG TCA GC R: CAA ACG AAG GCA CAA TGC CA
Fp- Sat48	48	Tandem	667	782	1669	0.15	0.33	68	+	F: TAA AAT GCG CAC CAT GTA GCT G R: AGG ATA CCT CCT ACG CAC CA
Fp- Sat84	84	Tandem	343	550	963	0.11	0.20	63	+	F: ATG GGA TGC AGT CAA AGG GG R: AAG TTT TGG GGC CAT TCC CT
Fp- Sat253	253	Tandem	372	0	77	0	0.015	100	+	F: ATG TGT GCA TGA GCT TTT GTT G R, CCG AGA CTT GAT TTT TGG GCT TT

Repeat type, monomer length, their proportion in genome, enrichment on Bs, Presence of FISH signals on Bs and primer pairs used for their amplification are indicated.

**Figure 2:**
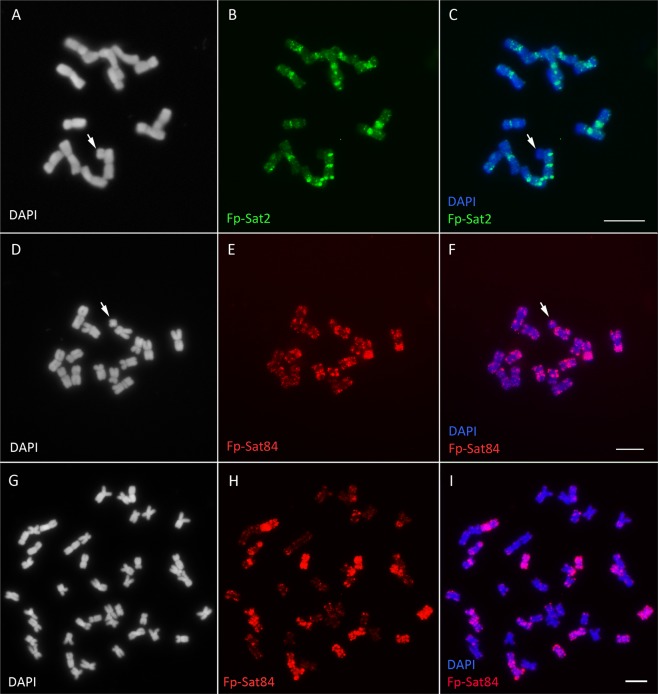
Localization of Fp-Sat2 and Fp-Sat84 tandem repeats on the *Festuca* chromosomes. **(A–C**) DAPI stained chromosomes and banding patterns of Fp-Sat2 (green signals) on *F. pratensis* chromosomes. (**D**–**F**) DAPI stained chromosomes and Fp-Sat84 localization showing overall distribution on the *F. pratensis* chromosomes (red signals). Arrows point to the B chromosomes. (**G**–**I**) DAPI stained chromosomes of *F. arundinacea* (genome FpFgFg’), distribution patterns of Fp-Sat84 repeat on its chromosomes and the resulting merged image. Scale bar = 5 µm.

**Figure 3:**
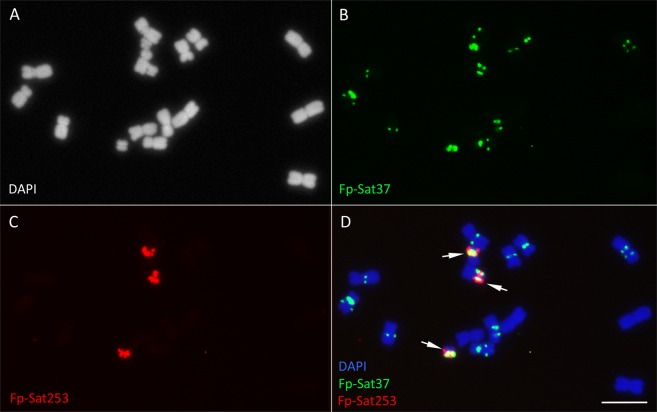
DAPI stained chromosomes (**A**), distribution patterns of Fp-Sat37 (**B**) and Fp-Sat253 (**C**) on the A and B chromosomes in a mitotic metaphase preparation of *F. pratensis* with 3 B chromosomes (arrows). The resulting merged image is shown in (**D**). Scale bar = 5 µm.

Labelled Fp-Sat2, Fp-Sat84 (Fig. 2) and Fp-Sat37 (Fig. 3) repeats produced hybridization signals on the A chromosomes. Only repeat Fp-Sat2 was not detected on the B chromosomes (Fig. 2C). Fp-Sat84 and Fp-Sat37 repeats also produced signals on Bs (Figs. 2F and 3). Fp-Sat37 produced stronger signals on the Bs compare with the A chromosomes (Fig. 3). Fp-Sat84 gave rather dispersed signals on all *F. pratensis* chromosomes although some signal clusters were detected (Fig. 2F). In order to test whether this repeat is species-specific, we performed FISH with the chromosomes of the allopolyploid species *F. arundinacea* Schreb. (2*n* = 6*x* = 42, FpFgFg’ genome); a species were *F. pratensis* contributed a subgenome^36,43,44^. In total, a subset of 14 of the 42 chromosomes showed Fp-Sat84-specific signals (Figs. 2H,I and 3) although the signals were not evenly distributed and some cross-hybridization occurred on a few other chromosomal regions possibly due to probe patterns itself or inter-subgenomic translocations (Fig. 2I).

FISH with the FP-Sat2 repeat resulted in chromosome-specific banding patterns in *F. pratensis* and proved to be a suitable cytological marker for chromosomes identification (Fig. 2C). BLAST analysis revealed that FP- Sat2 has 82% similarity with a middle repetitive sequence of *L. perenne* (accession AF063226.1) and 78% similarity with a cDNA from gliadin genes in *Triticum aestivum* (accession MG560141.1) (Table 1). Furthermore, Fp-Sat84 and Fp-Sat162 showed 98% and 96% similarity with satellite TR7 (accession JX624133.1) and TR15 (accession JX624136.1) respectively, which previously were identified from sequencing data of chromosome 4F^38^. No similarity was found for Fp-Sat131, FP-Sat37, Fp-Sat71, Fp-Sat75, Fp-Sat130, Fp-Sat159, Fp-Sat175, Fp-Sat236, and the B chromosome enriched repeats FP-Sat48 and FP-Sat253.

### Identification of B chromosome-specific repeats in *F. pratensis*

**Figure 4:**
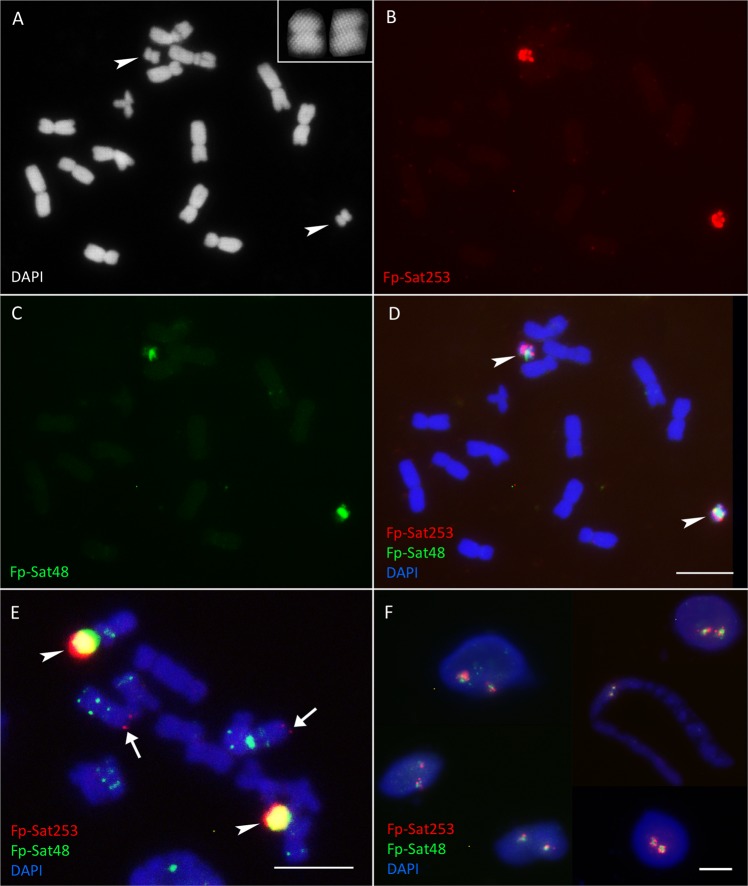
B chromosome painting in *F. pratensis*. (**A**) A DAPI-stained metaphase cell of a *F. pratensis* plant with two B chromosomes. Enlarged Bs are also shown in top-right corner of the picture; (**B**,**C**) B-chromosome labelling with the B-specific probes Fp-Sat253 and Fp-Sat48, respectively. Arrow heads indicate B chromosomes; (**D**) Combined picture resulting from merging A, B and C subfigures showing painted B chromosomes. (**E**) FISH using Fp-Sat48 (green signals) probe generated strong signals on Bs and faint signals on some of the A chromosomes. FISH also resulted in B-specific localization of Fp-Sat253 (red signals), although week telomeric associated signals on one of the A chromosome pairs (arrows) were detectable after an extended exposure time. Arrowheads point to the B chromosomes; (**F**) FISH on leaf interphase nuclei detecting the number of B chromosomes in *F. pratensis* plants. This picture shows the result of FISH using B specific probes Fp-Sat48 (green) and Fp-Sat253 (red) on nuclei from a plant having 2 Bs. Scale bar = 5 µm.

**Figure 5:**
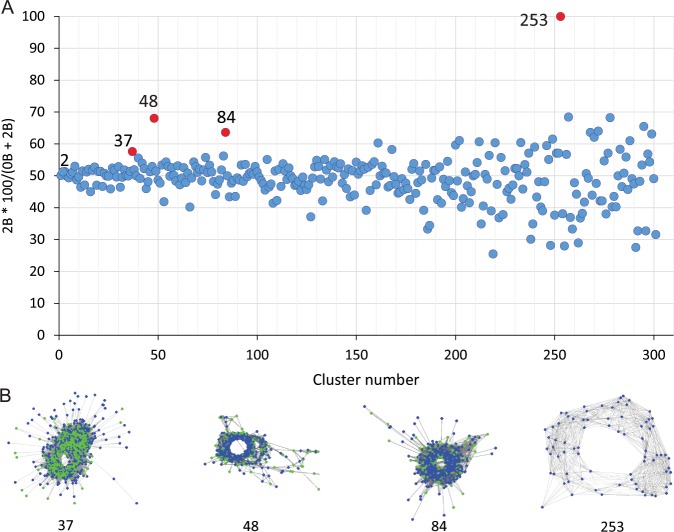
(**A**) *In silico* identification of consensus sequences of satellite DNA enriched in *F. pratensis* B chromosomes. Potential B enriched repeats (red dots) were identified by comparison of genomic proportions of individual repeat families in −B and +B genotypes. Dots on the plot represent individual repeat clusters. The vertical axis is the relative abundance in −B and +2B genomes indicated by the percentage cluster size in the +B genotype (harboring 2 B chromosomes) divided by its size in the −B (lacking B chromosome) genotype, resulting in a value of 50 for sequences with the same sized in both genotypes. (**B**) Graphical layouts (generated by TAREAN) of repeats potentially enriched in Bs.

**Figure 6:**
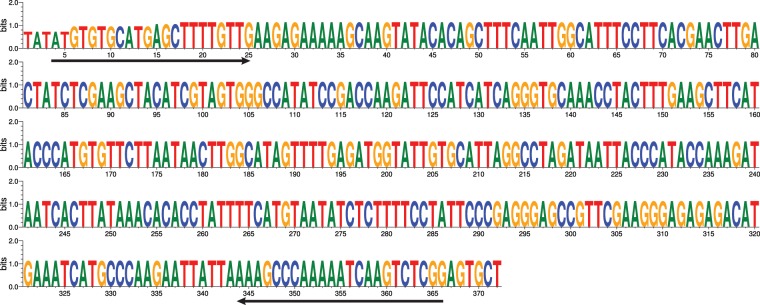
Sequence logos for the full-length consensus monomer of the B specific Fp-Sat253 (372 bp) satellite DNA in *F. pratensis*. This sequence was rebuilt from the most frequent k-mers (11-,15-, 19-, 23- and 27-mers). K-mer frequencies for each satellite is calculated from Illumina reads and is used to reconstruct their most conserved fragments. These fragments are then merged to create full-length consensus monomers shown here for Fp-Sat253. The height of the letters reflects the frequencies bases in the corresponding k-mers. The arrows indicate forward and reverse primer sequences used for the amplification of Fp-Sat253 satellites by PCR.

We identified plants harbouring 0B, 1B, 2B and 3B chromosomes. A similar number of Bs were present in various cells of both roots and leaves of each B chromosome containing plant (Fig. 4) so Bs might be retained in all the different plant tissues. The Bs can easily be distinguished in *F. pratensis* by the morphology, being roughly one third to one fourth the length of the A chromosomes and with a subterminal centromere (Fig. 4A). Comparative analysis in RepeatExplorer recognized the tandem repeats Fp-Sat37, Fp-Sat48, Fp-Sat84 and Fp-Sat253 with monomers of 347, 667, 343 and 372 bp, respectively as potentially B-enriched sequences (Fig. 5; Table 2). The full-length consensus monomer of the B-specific Fp-Sat253 (372 bp) is shown in Fig. 6, rebuilt from the most frequent k-mers (11-,15-, 19-, 23- and 27-mers) by TAREAN. The B-specific candidate repeats were amplified from the +B genotype by PCR, although all of these repeats could also be amplified by using the −B genotype DNA as template. Fp-Sat253 primers produced a rather clear distinct band using the genomic DNA of −B genotype but a smear product (with occasionally a faint band) using the genomic DNA of +2B genotype after electrophoresis of PCR products in agarose gel (Supplementary Figs. S1 and S2).

FISH showed a B-specific localization of Fp-Sat253 along both chromosome arms (Fig. 4B). Additional A-located signals were found from Fp-Sat253 repeat after an extension of the exposure time (Fig. 4E). Probe Fp-Sat48 generated strong signals on Bs and a few faint signals on some of the A chromosomes at normal exposure ranges (Fig. 4C; Supplementary Fig. S3). Fp-Sat253 repeat seems to be localized at the telomeric or subtelomeric regions while Fp-Sat48 concentrated at the pericentromeric regions of the B chromosomes (Fig. 4D). Fp-Sat37 satellite sequence produced strong signals on Bs, although it was available on the essential A chromosomes as well (Fig. 3). Among the 5 studies satellite repeats, FP-Sat2 was the only repeat that did not produce signals on the B chromosomes. Because of the difficulty of chromosome preparation due to the small size of *F. pratensis* roots, we were interested to test FISH on leaf nuclei to identify +B genotypes. FISH using both B-specific probes on nuclei isolated from leaf proved to be a fast and efficient way of screening the +B plants (Fig. 4F).

## Discussion

Low-pass Illumina sequencing of the genomic −B/+B DNA followed by the repeat characterization from the sequence reads using RepeatExplorer^41^ and TAREAN^42^ pipelines provided a comprehensive view about the types, abundance and organization of repetitive DNA sequences in *F. pratensis*. A low coverage sequence of 0.4x was more than enough to elucidate the repetitive sequence landscape. The chromosomal distribution of five different repeats was determined by FISH. The analysis revealed that 61% of the genome of *F. pratensis* is composed of repetitive sequences. Such a high proportion of repeats is commonly found in plants which also show a wide range of genome size^45^.

Transposons (with the majority being LTR retrotransposons), were the most predominant repeats. The abundance of retrotransposons could be related to their active mechanisms of self-proliferation throughout the plant genome via RNA mediates^46–48^. Among LTR retrotransposons, the frequency of Ty3-gypsy retrotransposons (32.97%) was three times as high as that of Ty1-copia (7.78%) in the genome. In *Avena sativa* and *Triticum aestivum*, Ty3-gypsy elements occupy 40% and 46% of the genomes, respectively, approximately three times more abundant than Ty1-copia elements in both species^49,50^. A high proportion of the Ty3-gypsy superfamily has also been reported for chromosome 4F of *F. pratensis*^38^. Based on FISH data, Křivánková, *et al*.^39^ reported Athila as a highly frequent retroelement in *F. pratensis* genome. Our data showed that Retand^51^, with the frequency of 17.28% of the genome, is at least three times abundant than Athila retroelement (Fig. 1).

Satellites are believed to be amplified via uneven crossover and slippage replication^52,53^. These highly repeated tandem arrays provide useful markers for the analysis of chromosome evolution. According to our data, a relatively high number of satellite repeats represent 4% of the *F. pratensis* genome (Table 1). Satellite sequences compose 1% of *Capsicum annuum* L (3.1 pg/1 C)^54^, 3% of the genome in *Glycine max* L. (1.1 pg/1C)^55^, 2% of the genome in *Avena sativa* (12.85 pg/1C)^49^ and less than 0.1% of the genome in *Passiflora edulis* (1.26 pg/1C)^56^.

Křivánková, *et al*.^39^ established a FISH -based karyotype of *F. pratensis* based on different tandem repeats and rDNA probes. We also identified a tandem repeat (Fp-Sat2) that produce distinct banding patterns on all the A chromosome pairs although it was the only repeat not available on the B chromosomes. Repeat Fp-Sat84 showed a rather dispersed distribution along *F. pratensis* chromosomes and labelled the corresponding subgenome in *F. arundinacea* 6*x*. This evidence confirms previous conclusions that *F. pratensis* is one of the parental species of *F. arundinacea*^43,44^. Although Fp-Sat84 didn’t show sequence similarity to LTR retrotransposons, dispersed patterns of this repeat suggests that Fp-Sat84 may has evolved from retrotransposons or retrotransposition may has played a role in its evolution and distribution throughout the genome^57,58^.

Fp-Sat48 and Fp-Sat253 were identified as the first B chromosome-specific repeats of *F. pratensis* (Fig. 4). Fp-Sat37 also showed to be a B-enriched satellite sequence, but it was also abundant on some of the A chromosomes mainly as pericentromeric bands (Fig. 3). The identification of weak A chromosome-located signals and the presence of PCR amplicons in −B samples of four of the five studied repeats suggests that the Bs of *F. pratensis* resulted from the A chromosome complement of the same species. A similar evolutionary link between A and B chromosomes was found for a number of different species^33^. As an example, Bs in *Plantago lagopus* are composed of mainly 5S rDNA-derived sequences and various types of repetitive elements^59,60^. We did FISH using 5S and 45S rDNA probes on a +B genotype of *F. pratensis* but no trace of the corresponding signals was observed on B chromosomes (data not shown). In rye and *Ae. speltoides*, the Bs are enriched in organelle DNA^61,62^. However, based on RepeatExplorer analysis, abundance of organelle DNA sequence were not different between −B and +B DNA genomes of *F. pratensis* in the repsent study.

## Conclusions

We analyzed the repetitive DNA content of *F. pratensis* by low-pass Illumina sequencing of the genomic −B/+B DNA followed by the repeat characterization from the sequence reads using RepeatExplorer^41^ and TAREAN^42^ pipelines. 61% of the genome is composed of repetitive sequences and transposable elements are the major component of the genome. 4% of the *F. pratensis* genome is composed of satellite repeats which were partly harnessed as cytological markers for chromosome identification and genome analysis in the genus. Furthermore, the identified B-specific tandem repeats can be used as a marker to trace the dynamics of supernumerary chromosomes to provide direct insight into the cellular basis of the B chromosome drive mechanism in *F. pratensis* in future.

## Methods

### Plant material

An ecotype of *F. pratensis* (2*n* = 2*x* = 14) originally collected from Iran and received from the Research Institute of Forests and Rangelands of Iran, showed to carry B chromosomes. Single seeds of *F. pratensis* were inspected by chromosome counting using a dropping method as described in Abdolmalaki, *et al*.^63^ for the presence or absence of B chromosomes. For this, one or two primary root tips of each single seed were analyzed and the corresponding seed was transferred to a pod to grow. We could identify plants harbouring 0B, 1B, 2B and 3B chromosomes. DNA samples were extracted from the leaves of *F. pratensis* individual plants possessing 0B (−B) or 2B chromosomes (+2B) and sequenced using the Illumina HiSeq 2500 system at low coverage.

### Sequence analysis and identification of A- and B-specific repeats

Identification of *F. pratensis* repeats specific to the essential A and supernumerary B chromosomes was performed by similarity-based clustering of Illumina reads using the RepeatExplorer pipeline^41,55^. The CG content of the reads was estimated from the *F. pratensis* genomes using the Illumina R1 and R2 output files with the FastQC tool. All sequences were filtered by quality with 95% of bases equal to or above the quality cut of value of 10. Paired reads were joint into a single FASTA file using FASTA interlacer. The clustering was performed using the default setting of 90% similarity over 55% of the read length. Comparative clustering analysis for the identification of B chromosome specific repeats were performed after assigning 0B and 2B prefix codes to the respective reads of −B and +2B genotypes. Each set of data was down-sampled to 500000 reads to represent 0.1 coverage of each genome followed by their concatenation into a single data set. The analysis allowed for the identification of repeats enriched on B chromosomes. Monomer lengths and consensus sequences of the identified satDNA families were determined using TAREAN^42^. WebLogo^64^ was used to generate de Brujin graph of the reconstructed consensus sequences of satellites predicted by TAREAN. Consensus monomers of satellites were used to design PCR primers for amplification of satellites including Fp-Sat2, Fp-Sat37, Fp-Sat48, Fp-Sat84 and Fp-Sat253 (Table 2).

### Preparation of FISH probes

Genomic DNA of −B/+2B genotypes were used as template in polymerase chain reaction (PCR). The PCR mixture contained 25 ng template DNA, 5 pmol of each primer, 2.5 mM of each dNTP, 2.5 mM MgCl_2_ and 0.5 U *Taq* polymerase. Amplification was for 5 min at 94 °C, followed by 30 cycles of 45 s at 94 °C, 45 s at 58–61 °C (depending on primers), 90 s at 72 °C and a final step of 7 min at 72 °C. PCR products were purified by ethanol precipitation. PCR products were labelled with Atto-488-11-dUTP or Atto-550-11-dUTP using a nick translation kit (Jena Bioscience, Jena, Germany), recovered by ethanol precipitation and used as a probe in FISH.

### Slide preparation for FISH

Seeds were germinated on moist filter paper in Petri dishes for 3–6 days at room temperature. In order to stop cell division at metaphase, root tips of about 1–1.5 cm length were cut and placed in microtubes punched at the top. Roots were subjected to nitrous oxide (N_2_O) gas at 10 bar pressure for 2 hours. Treated roots were fixed in ice-cold 90% acetic acid for 10 minutes, and then transferred to 75% ethanol and stored at −20 °C until use. Roots were washed in ice-cold water, followed by 0.01 M citrate buffer each for 10 minutes. Meristematic part of the roots (about 1 to 1.5 mm of the root tips) were cut and placed in a microtube containing 30 µl enzyme mixture (0.7% cellulase (CalBiochem 219466), 0.7% cellulase R10 (Duchefa C8001), 1% cytohelicase (Sigma C8274) and 1% pectolyase (Sigma P3026) prepared in 0.01 M citrate buffer, (0.01 M citric acid and 0.01 M sodium citrate, pH 4.8). Root tips were digested at 37 °C for 60 to 90 minutes and slides from digested root tips were prepared using dropping method according to Abdolmalaki, *et al*.^63^. The slides were fixed in 4% paraformaldehyde in 1× PBS (3 mM NaH_2_PO_4_, 7 mM Na_2_HPO_4_, 0.13 M NaCl, pH 7.4) for 10 minutes at room temperature, followed by washing in 2× SSC (0.3 M sodium chloride, 0.03 M sodium citrate, pH 7.0) and dehydrating in 96% ethanol.

For the preparation of nuclei, a small leaf of approximately 2 cm^2^ in total, from each *F. pratensis* plant was chopped with a sharp razor blade in 0.5 ml 45% acetic acid. The nuclei suspension was filtered through a Partec (Partec, Münster, Germany) 30 µm nylon mesh filter. 10 µl of the nuclei suspension was dropped on a microscopic slide and briefly heated to dry for subsequent FISH.

### Fluorescence *in situ* hybridization (FISH)

Twenty µl of hybridization mixture was placed on each slide and covered with a plastic coverslip. Slides were then denatured at 80 °C for 2 minutes on a hot plate. Hybridization mixture contained 2× SSC, 50% formamide, 20% dextran sulfate, 1 µg sheared salmon testes DNA and 20–30 ng of each labeled probes. For hybridization, slides were incubated in a humidified plastic container at 37 °C. Coverslips were removed and slides were washed in 2× SSC for 20 minutes in a water bath at 56 °C. Slides were dehydrated in 96% ethanol and dried at room temperature. A drop of Vectashield mounting medium (Vector Laboratories) containing 1 µg/ml DAPI (4′, 6-diamidino-2-phenylindole) was added to each slide as counterstain and a glass coverslip was applied. Slides were inspected with a fluorescence Olympus BX51 microscope (Olympus, Japan) and images were captured, using a DP72 digital camera (Olympus, Japan).

## Supplementary information

Supplementary information
