# Supplementary material for: Repetitive DNA landscape in essential A and supernumerary B chromosomes of Festuca pratensis Huds
Source: Sci Rep. 2019 Dec 27;9:19989. doi: 10.1038/s41598-019-56383-1 (PMC6934454; doi:10.1038/s41598-019-56383-1)
Supplement: Supplementary file 1 — Supplementary information [file 41598_2019_56383_MOESM1_ESM.pdf]

Article title:

Repetitive DNA landscape in essential A and supernumerary B chromosomes of *Festuca pratensis* Huds.

Authors:

Rahman Ebrahimzadegan

Andreas Houben

Ghader Mirzaghaderi

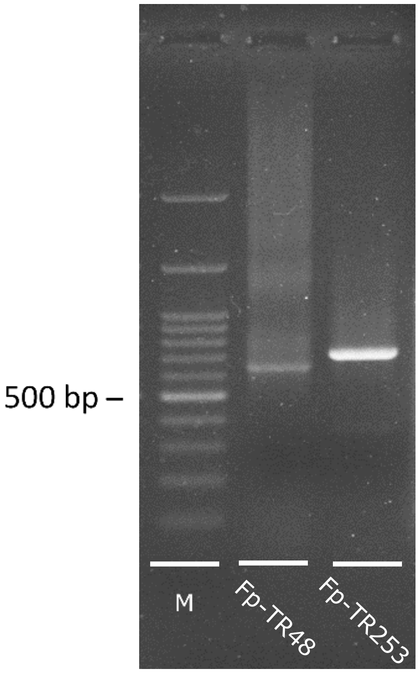

Figure S1. PCR amplification of Fp-TR48 and Fp-TR253 repeats from *F. pratensis* genomic DNA of a +2B plant.

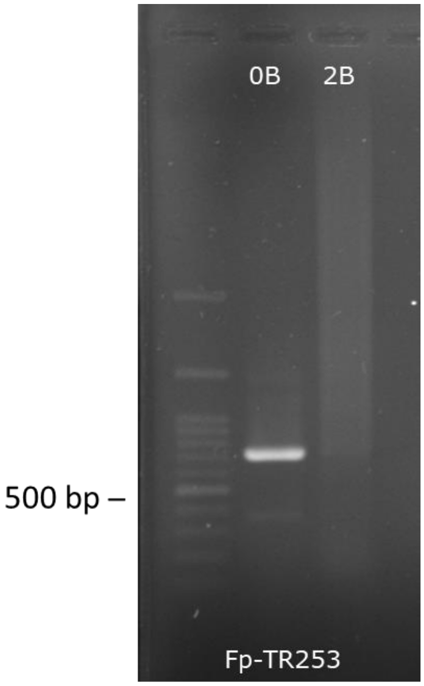

Figure S2. PCR amplification of Fp-TR253 repeats from *F. pratensis* genomic DNA of 0B and +2B plants.

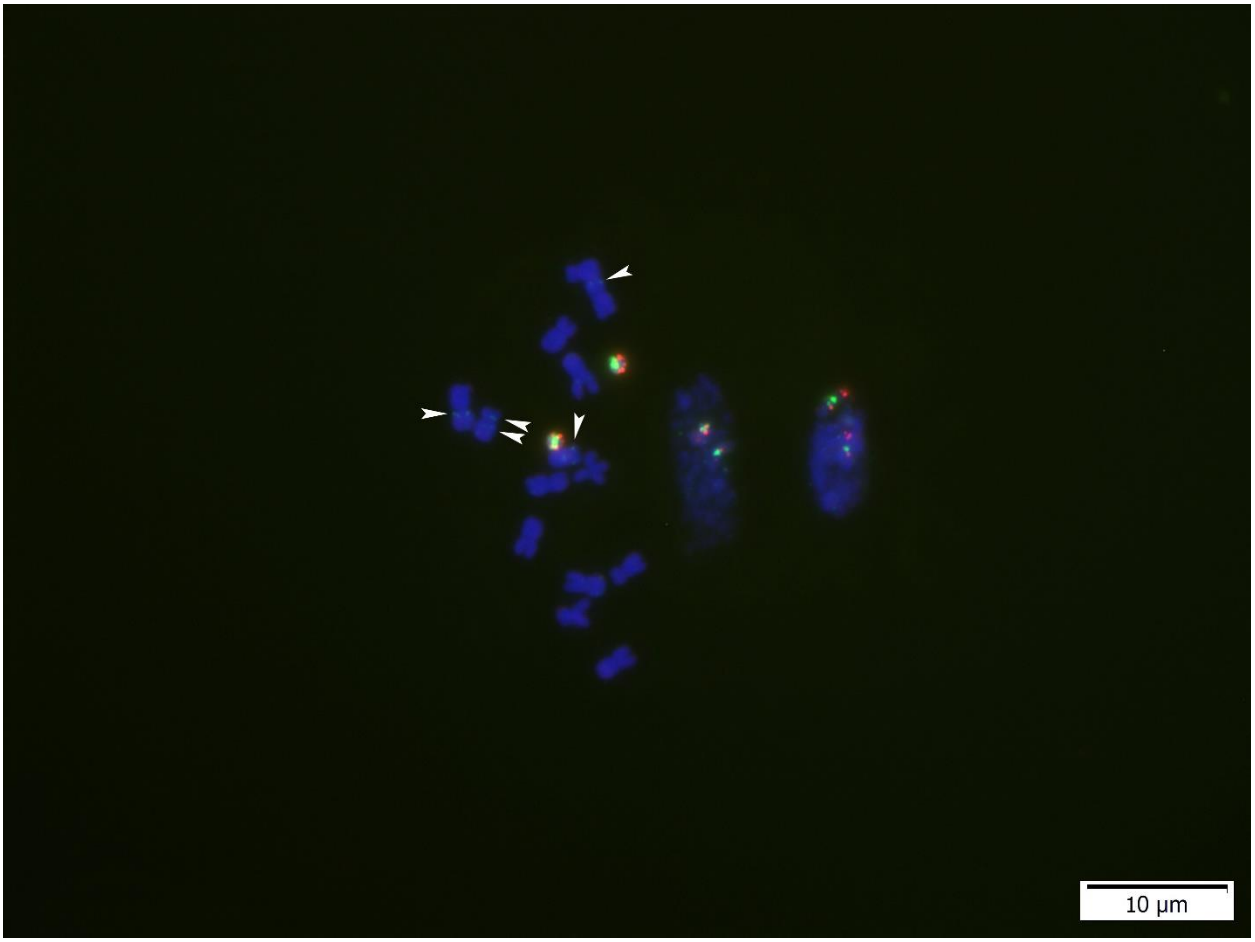

Figure S3. Distribution of the B-specific probes Fp-Sat48 (green) and Fp-Sat253 (red) on B chromosomes of *F. pratensis*. Arrowheads indicate additional faint signals from Fp-Sat48 probe on the standard A chromosomes.

Table S1. Comparative analysis of the genomic repetitive clusters in +2B and –B genotypes of *F. pratensis* along with the monomer counts for clusters.

Cluster #	0B	2B	Cluster #	0B	2B	Cluster #	0B	2B	Cluster #	0B	2B
1	3102	3118	126	357	319	251	33	44	376	19	24
2	2713	2860	127	423	250	252	33	44	377	24	19
3	2327	2458	128	329	320	253	0	77	378	10	32
4	2315	2308	129	301	337	254	47	29	379	24	18
5	2310	2256	130	281	342	255	54	21	380	26	16
6	2172	2224	131	286	322	256	32	42	381	12	30
7	2045	2148	132	310	298	257	23	50	382	25	17
8	1896	2141	133	350	255	258	46	27	383	29	13
9	2064	1964	134	266	327	259	48	24	384	23	19
10	1894	1872	135	277	298	260	37	34	385	22	20
11	1975	1711	136	282	262	261	39	31	386	19	23
12	1734	1846	137	251	286	262	49	20	387	19	22
13	1888	1686	138	268	264	263	43	25	388	23	18
14	1708	1794	139	240	287	264	42	26	389	23	18
15	1641	1784	140	247	277	265	39	28	390	20	21
16	1857	1521	141	242	268	266	33	34	391	17	24
17	1599	1716	142	229	267	267	34	33	392	18	23
18	1530	1712	143	240	219	268	24	42	393	24	17
19	1697	1511	144	226	230	269	37	29	394	19	21
20	1547	1633	145	234	200	270	25	41	395	23	17
21	1542	1632	146	195	213	271	34	31	396	15	25
22	1681	1474	147	212	163	272	23	41	397	15	25
23	1539	1589	148	178	190	273	37	27	398	21	19
24	1516	1515	149	201	158	274	37	27	399	22	17
25	1501	1497	150	163	194	275	29	35	400	17	22
26	1619	1376	151	174	178	276	39	24	401	27	12
27	1416	1563	152	166	177	277	27	36	402	23	16
28	1434	1540	153	154	175	278	20	43	403	19	20
29	1488	1467	154	151	164	279	37	25	404	9	30
30	1375	1544	155	189	122	280	35	27	405	16	23
31	1563	1354	156	139	155	281	37	25	406	18	21
32	1470	1444	157	155	129	282	31	31	407	17	22
33	1397	1507	158	123	142	283	25	35	408	23	16
34	1433	1444	159	118	141	284	32	28	409	18	20
35	1429	1428	160	134	120	285	29	30	410	20	18
36	1380	1460	161	100	152	286	26	33	411	14	23
37	1193	1624	162	125	125	287	32	27	412	17	20
38	1333	1440	163	115	130	288	23	36	413	21	16
39	1385	1388	164	116	120	289	30	29	414	16	21
40	1210	1516	165	109	113	290	31	28	415	15	22
41	1381	1338	166	116	101	291	42	16	416	19	18
42	1234	1449	167	106	94	292	39	19	417	13	24
43	1274	1394	168	83	116	293	30	28	418	26	11
44	1429	1217	169	100	99	294	27	31	419	16	21
45	1344	1248	170	100	90	295	20	38	420	15	22
46	1210	1276	171	103	83	296	39	19	421	20	17
47	1164	1312	172	98	80	297	25	33	422	25	12
48	782	1669	173	90	87	298	26	31	423	12	24
49	1169	1240	174	96	81	299	21	36	424	14	22
50	1199	1139	175	92	84	300	29	28	425	23	13
51	1222	1115	176	95	81	301	39	18	426	17	19
52	1087	1247	177	101	75	302	29	27	427	12	24
53	1355	978	178	80	87	303	29	27	428	26	10
54	1051	1252	179	77	90	304	29	27	429	15	21
55	1063	1199	180	91	73	305	40	15	430	25	11
56	1131	1128	181	85	78	306	23	32	431	14	22
57	1056	1170	182	78	83	307	17	38	432	16	20
58	1106	1112	183	82	78	308	32	23	433	10	25
59	1125	1053	184	96	62	309	26	28	434	26	9

60	1003	1140	185	71	82	310	32	22	435	21	14
61	1111	995	186	102	51	311	32	22	436	14	21
62	1065	1037	187	97	51	312	28	25	437	18	17
63	967	1074	188	74	72	313	33	20	438	24	10
64	1055	971	189	69	74	314	26	27	439	26	8
65	986	1021	190	68	73	315	21	32	440	20	14
66	1192	802	191	81	60	316	22	31	441	8	26
67	1007	981	192	66	74	317	29	24	442	15	19
68	873	1042	193	82	58	318	19	34	443	19	15
69	897	1009	194	71	68	319	21	32	444	22	12
70	893	934	195	72	63	320	29	24	445	16	17
71	859	936	196	60	72	321	31	22	446	11	22
72	921	866	197	68	64	322	27	26	447	20	13
73	801	863	198	73	59	323	19	33	448	14	19
74	732	923	199	73	57	324	30	22	449	19	14
75	799	834	200	52	77	325	24	28	450	15	17
76	760	870	201	66	62	326	34	18	451	20	12
77	785	826	202	49	77	327	25	27	452	12	20
78	830	774	203	73	49	328	26	26	453	24	8
79	888	702	204	68	51	329	22	29	454	18	14
80	831	749	205	58	61	330	20	31	455	20	12
81	792	754	206	60	53	331	16	35	456	17	15
82	737	795	207	66	47	332	25	26	457	19	13
83	665	855	208	61	50	333	35	16	458	14	18
84	550	963	209	55	55	334	25	25	459	17	15
85	740	741	210	54	55	335	23	27	460	17	15
86	828	636	211	49	58	336	27	23	461	10	22
87	750	684	212	42	65	337	28	22	462	15	17
88	734	695	213	69	37	338	31	19	463	9	23
89	787	607	214	63	43	339	30	19	464	20	12
90	696	680	215	52	53	340	25	24	465	19	13
91	696	660	216	53	51	341	23	26	466	12	20
92	618	709	217	44	57	342	26	23	467	18	13
93	661	618	218	61	39	343	26	23	468	18	13
94	585	676	219	73	25	344	26	23	469	18	13
95	632	627	220	38	58	345	27	22	470	25	6
96	616	635	221	50	45	346	24	25	471	21	10
97	586	614	222	60	35	347	26	22	472	17	14
98	592	605	223	42	53	348	21	27	473	11	20
99	539	607	224	59	36	349	29	19	474	16	15
100	558	586	225	45	48	350	29	19	475	15	16
101	568	560	226	50	41	351	28	19	476	19	12
102	579	546	227	49	42	352	26	21	477	14	17
103	578	522	228	51	38	353	20	27	478	15	16
104	596	499	229	46	41	354	20	27	479	8	23
105	485	597	230	41	45	355	16	31	480	14	17
106	572	498	231	41	45	356	25	22	481	18	13
107	543	491	232	43	43	357	35	12	482	16	15
108	568	402	233	34	52	358	21	25	483	16	15
109	450	479	234	46	39	359	26	20	484	19	12
110	532	390	235	39	45	360	24	21	485	19	12
111	436	474	236	41	43	361	19	26	486	14	16
112	471	413	237	36	48	362	25	20	487	13	17
113	397	462	238	58	25	363	20	25	488	21	9
114	406	432	239	54	29	364	20	25	489	19	11
115	426	407	240	34	49	365	21	24	490	12	18
116	382	423	241	47	36	366	29	15	491	18	12
117	401	402	242	43	40	367	21	23	492	19	11
118	406	392	243	49	33	368	24	20	493	19	11
119	423	375	244	50	32	369	24	20	494	17	13
120	409	369	245	42	39	370	32	12	495	20	10
121	395	370	246	32	47	371	23	21	496	19	11
122	415	345	247	35	43	372	24	19	497	22	7
123	367	362	248	56	22	373	13	30	498	16	13

124	374	333	249	30	48	374	22	21	499	13	16
125	382	322	250	48	29	375	25	18	500	16	13

Table S2. Consensus monomers of the identified satellite sequences in *F. pratensis* determined by TAREAN analysis. Five sequences including B chromosome specific Fp-TR48 and Fp-TR253 used to design the primers as described in Methods.

Cluster	Name	Genome Proportion [%]	Size real	Satellite probability	Consensus length	Connected component index C	Pair completeness index P	Kmer coverage	|V|	|E|	Consensus sequence
2	Fp-Sat2	1.1	5573	0.741	317	0.952	0.887	0.621	5570	3780000	TGAAAAACCAAAGCACATAGTCTTCTTATGTCATATAGTAAGCATTCAAGTTGATAAACAAGGTCTAGACATATTCAAACCAGAAAAATCATGTATCTACCCCCTAACCCTAAACTCTGTCTTATGAAGTCAAGCATGAAGAGAAGTGGTTTTGGGTTTCAAACATGGAAATTTCAAAAACCTCCCAAAAGCTTCATTTTGGAGTGACTAAGAAGGATCCAGGCTTACTTTTTCATTTTCATGTTTTAAACTTGTTTAAATCTTGGTCAAACCTAGGATTTGACCAAGATTCAAATGGGCATAAAAATTCAAAAAAA
48	Fp-Sat48	0.49	2451	0.908	667	0.96	0.918	0.587	2451	431000	CAATACTAAAATGCGCACCATGTAGCTGCTTCACAAAAAAAGGCCTTCCGGCACCCCGAAAATGGAAAAACCGTCGCCCGTGGTTCGGATTGGAAATCCGCGACCGGGGCCTTGCTTCCCATCCTAAGGCCCACGCACGTGCCAAATATGGCCTCGTTCCGACAAACTATGTGGTGAAACGGGCCGTTTCCTACTCATTTCCCCTAAAAGCCATAGAACTCCGGACGTGATAGCCCTATTCGTGAAGGGTTTTTCAAGATAATTGCCGTATCCCAATTCCGTCTTTTGCAGTGGTTACTAGGACACATAAAATGACGCCACGCGGCTCCGCTCGACTTTTTGGCTCCGTTTACTTTGCCAACTGTGCAAAACGGGGCCGCCGGGACATGCGGTATGGCCGTACCGGGCGCGCGGGTGCACCCGTCCGCCAACGCGCCAAACGGAAAACATTTAGCACATAAAATGACGCGTCCTAGACCTAAAAACATTTTTCCCTCCATTTATTGAGCGAAGGAAAGTGTGGCCCCAGTTCAAATCCGGTCAGTTTCCAGCGGATTCGGCGGGACACCGCAGGAAGCGGGAGGGATTCCCAGATGGCTACCGCGCGCATATGGCATGTGATAGGTGGTGCGTAGGAGGTATCCTACCGCTGCGCGGAGGTCTCGCG
131	Fp-Sat131	0.12	608	0.748	659	0.964	0.848	0.595	608	19100	GGTACCATTTGGAACCGTATCTTTTGTATCTCATTCACAAAATAAACCTATTTCGTCATTTAGAAAATGAAAAATGATTTTCTTTATACCAAAAAGTTGAAAATTCTAGTTTGCAACATTGTTGGGAATGTCAAGATGCACCACCATGCGCAATTCGGGCACGTTATAACAAGCTATGCCACAAATATGGCCATAACTATGTCATTTGGCTTGAAAGCTATGAATCTTCGTACACGATAGTCCGTTCTGTGAAGACATTTTGTTTTTTACTACCCTATTACAGGTTTCTTATTTTCACTGTCAACTAGTACACATATTAGCACTCCATGCGAAAGGATTTCATTTTTTGAGGTTTTCTTCATTTTCTTTTATTTATTTCAAAACCGGGTCAAAATGGTCGACATGATTATTCATAGAAAGGGGTTGAATATTTCAGCACTATCATCGGTTTCACTATGTAATAAGTAATATGTATCTAAATAAGATTTTTGGGGACTTGTAGGACCAAGTTATAACACACCTACAGTTCAAATTTGAATTACTTTCAATAAATCGGCATAAAGTCATTTAAATGCGCGAAACTAGCTACGAATCATTAAAATTCGTAATAGTTGGCAATTGCCACTGAAATGTGGCCTACTTTACCTGAAAAATGTAGA
162	Fp-Sat162	0.05	250	0.815	346	0.944	0.908	0.738	250	8210	AGCCCACTTGGTGGCCTGGGTAGAGCCGTTGTGCCCAGAAACCATGTGTGCGGGCTTCCGTGAGGTAGCTAGGCAAGGAGCACATGCATTCTTGCGTGGATGGTGATGTAAGTGGTGTTTGCATGCCATGGTGAAGCAGTTCCCAAAAGATGGGATCACTATGCACACACACCGTCAGACGATCCACTGTTTCGACACCTTTTTCTGACGAGAACCCTATATTTGAGGGGTCTACACCACATGAACATGGGCATTCACACATGCAACATGCAAAACCCATGGCTTGAAGTGGGTGGAATGACATGGTGCACCAGTGCCCACAACATGGGGTCATCCCAGGGTTGGC
253	Fp-Sat253	0.015	77	0.948	372	0.987	0.925	0.8	77	987	TATATGTGTGCATGAGCTTTTGTTGAAGAGAAAAAGCAAGTATACACAGCTTTCAATTGGCATTTCCTTCACGAACTTGACTATCTCGAAGCTACATCGTAGTGGGCCATATCCGACCAAGATTCCATCATCAGGGTGCAAACCTACTTTGAAGCTTCATACCCATGTGTTCTTAATAACTTGGCATAGTTTTGAGATGGTATTGTGCATTAGGCCTAGATAATTACCCATACCAAAGATAATCACTTATAAACACACCTATTTTCATGTAATATCTCTTTTCCTATTCCCGAGGGAGCCGTTCGAAGGGAGAGAGACATGAAATCATGCCCAAGAATTATTAAAAGCCCAAAAATCAAGTCTCGGAGTGCT
37	Fp-Sat37	0.56	2817	0.679	347	0.923	0.927	0.51	2815	896000	GAACATGCCTAATTGCCGTCAGCGCATGTGCGTGTAGCCGGACGCACCCGGGGGTACAAAATTGGTTCAAACAACACCCCACCATGATAGTGTACACACCATGTGGACACACACAATTTTTTGGGTCATTCCCACCCTCCGAGGCT

											CGCTTTCCAGGGAAACCCTAATCCATTGACCGTGCGGTCTACAACATTGACTACACCCCAGCGGCTGTGCCGC<br>GGTGGCATTGTGCCTTCGTTTGAGCTTGGTTAGGTCATGGGGACATGTGGTGGCAAGGATTGATCCATACTA<br>TCTCAAGATTCCCTCCGGTTCACCCCACCGGGGAGTTCCACCCTATACGTCTACTA
71	Fp-Sat71	0.36	1795	0.0184	956	0.719	0.681	0.262	1795	30400	CATGATGCTACCCTGAATTTGTGGGACCCCAAAAAACTTGCAAATACGAGCGATGCATCCAAAAGAAACACTC<br>GTCCCCTCCCCCCATGATTTTGACCTAAATTTGAAATCAAACAGAAGGTGGTTAGAAAAAATCGAAATTGGCA<br>AAGCAAAGCATGAAATCATAAATTTGTGATGCACTCTAGCTATGAAAAGTACCAAACAATGCATACCAAAATG<br>ATTTTTTAGAGGAATACTAGTTCAACTTATTTAATTTTATATTAGTAGGCCAAAATCGAACCCCGTAGTTGGAT<br>TTTTTTGAACTTGATCTGTAGTACTGGGCAAAACCCTAGTTTAACCTATTTAATCATAGATTCGTAGGTCGATTT<br>TTTTACGTTTTTATTATTATTACTATGCAGCACATGTGTGTTTGCCCGTCCAGTGAAACTCGGAGGAAGAGGG<br>ATCACACGGAAGGAGACGAAGGGAGAGCAGTCACGGGGCCCCTCTTATTTATGGTGTCTCCCCTTTTTCGGG<br>TGATGTTGACTGTTGTGCAGGGTTTGTCAGTGCGCAGGAGGTGCGAGCGAGCGAGGCCGAGAATGAGAGA<br>GATGTTTTTTTTGTGAGAGGGACAACCGAAGGATCCTGAAGGACCCAGAGACTTCCAATACGCATCCTGATG<br>CGTCTTCTCTTGACAAAGAAGATGGCTGGGAGTTTGCCTACCTACCGCACGTGACTTGTGCTCACTGAAGTGT<br>GGGACCTCACGCATGGGAACCACACGTAAGTGACAGACCTGTTGGTGTAATAACTCGGGTGATTATTATACT<br>GTATTAGTACAAAGTTTCCTATGGATTCAAGGGTAGGAAAGCCAAGTTAACTCATCTATCAATTGGTACATTTT<br>GGTTATATTCAAAATTTTACTTGAAACTTGTTGGTCATGCCATTCTACTACTCGCCTAACACTTTTTCCGCGCGG<br>GAAGGGGGG
75	Fp-Sat75	0.33	1633	0.022	136	0.734	0.825	0.444	1633	83300	TACAATAAAAGTTTCTTTTCTTGGATTATTTTTGGCCCCTGATATTTGGTCACTTGCCTTTTTCTTTCGATCCCAT<br>GCCGCCTTTGAAACGTTGAGAACGCTGCTGCCAAATGGGACCCGCATGTCATCCTCTATG
84	Fp-Sat84	0.3	1513	0.531	343	0.929	0.891	0.739	1513	329000	GGTGTGTCCGGCTTCTCGGACAGGGGGTTCCTACACTTAGGCAGATTCTGCATGTATAGGGGGGAACTCCCT<br>CGGAGGTGAACCGGAGAGAACCTTGGGATCATATGGGATGATCCTTGTTTCCACATGTACCTATGACCTAACC<br>AAGCTCAAATGGAGGCATATGCCCACCGGGGGACCCCCGATGGGATGCAGTCAAAGGGGTAGACCGCGCG<br>GTCAACAGAACTAGGGTTTCGTCAGAAATCGAGCATCGGATCTAGGGAATGGCCCCAAAACTTGTGTGTGTC<br>CACATGGAATGCACAAAAGTGATTTGAGATGGTTTTATATCCAAGGCTACCCCCCA
124	Fp-Sat124	0.14	707	0.612	173	0.928	0.916	0.492	707	55100	CTAAAACCCTTGTTTCGGCACCTCCCCACCATGGGATCACCATGAAACACACAACCAACCTAGAAAACCCAAG<br>GTAGCAAGTGTGGGGCATGCCACCATGCACAAGTGTGCCAAATATTGGCACCATCCAAGGTGGGTTTCTATG<br>TTTTAGGCCAATTAGACCCCTTTTGACC
130	Fp-Sat130	0.12	623	0.0344	305	0.788	0.653	0.485	623	31100	GAGGCAGTAGGATCGGAAGTTTTTCTTGTATGGTTATTTTATTTCAAGGATTGATTTCCGTACTAGCTAGTATT<br>CTTCCAAACAAACTTTCAATTACGCTAAATTTCATAGGAAATCTAACGCATATCCACCCATATGTATATACCTCT<br>ATTTCATGTGGTGGAATCAAACAAACTATGGTTAGGGTTAGGGTTATAGATAAACCTTATAGTGCTCCGATTC<br>ATATTACTTGATGATAATATAGATGTATCTGGACACATTTTAGTTATAGATACATCCATTTTAGCATCAAGTAG<br>TATGAATCA
133	Fp-Sat133	0.12	605	0.0746	175	0.86	0.885	0.573	605	48800	CAAATATAGGGTTTTAGGCCAAAACATGCACTTCTTGGGCAAATCAAGTAGCTGGGGATGCCAGGGTGAGGC<br>CACACTTGGCACATGGGTGCACCATTGCATGCCCCACACTGCTACCCTTGGCATCTACATGAATAATCTGGGG<br>TAGACCATGGTGAGTTGCACCCAGACACCC
159	Fp-Sat159	0.052	259	0.057	340	0.869	0.786	0.603	259	7730	TTGCTTTGGTTTGTTTAGGACATATGTACATGGAAACCATAGGTTGGGCATACTTTACCATAAAATAGGCTCC<br>GTTTGAGCCGTAGCAGGAGTTTCCACATACAGATCCTGGATTTTACCAAGTGCTAGCCCATGTATGCGGAAAT<br>CGTCAACGCCGGGTACATGCAAGGTTAGCCAAACCTTACATCACACTTGTACACATATGTTAGGGACAGATGC<br>AAGTTTTCCAGCGATTCCGACGCTCCGGTGATCTGTATCTTGGGAAACCCTAGGGCGGGGACCCCGCAGATCT<br>ACCCCTATAGCTTTCTCCGTGCGAGGGTTTACCAATGGACTTTTTTAT
161	Fp-Sat161	0.05	252	0.0952	660	0.905	0.8	0.673	252	4280	TGTGAAGCAACCACCAAATTAAAGTTTCACATTGGTACCAACACATTTTTCAAAAATACTAGACCACCTAAGAT<br>GGATAATTTCCCAACCGGTGTATCTGGAACCTTTCCGTCCACCCCTCGTGAAAAAGACAAATTCCTGCCGAATC<br>GGTAGGAGGCCGGCCAGATTTGAACTACAGGCACATGATATAATGCTCAAGATTTTTTGTAAAAATATTTATT<br>GGGTTCACAACATGCTATTTCATCAGAGATCCAGTGCAAGTTTAGCGAAACTCCGCCCCGAGCGAAGGATCTT<br>CATGCCCGCCGTTTTGAATATAGTTTGAAACGAGCATAAAAAATTCAAAAACGATCCAAACAACGCGAAACCT<br>TCGCGTGCTGTCATATTATGTGTCATAGTTGCAAGGAAAAATAATAAACTTGGATAATTGCATACATCACAAA<br>AAATCCTTCACAATATGAGCTATCATGTTCGAGGTTTCATGGCATTTAGGTCAAACGACCAATGTTACTGATAG

175	Fp-Sat175	0.035	176	0.0424	842	0.795	0.725	0.315	176	949	AATCGCGACATAGTTTGTGATAATGTGCCCATATTGTGCACACATGTGCATCTTGGGATGTCTTACGATGTTG<br>CAGGAGGAAGTTTTCCTTTTCATCGCACGAAAAAGCCATTTTCCATTTTCGAGGTGCCGAAAAGGGGGTGTTT<br>TCAGCTCCGCTGCTGCTGCCTTGCAGACCAATTTAGCTCTAGATGCTTCTCTGCCCGCTTCGAAGGATTTATCC<br>TAGGCAAAGGGATCCCTCAAGCTTGGGCTTTTGTAGCGTTGAGCCTTTCGTGCGGTGTCACCGAAGTATAGTC<br>TCTTTGTACATGTAAACACAAAATAAGCTAAAGTTAACAAATCCAGTTTCAGATAACCATAAAGCCCCACAACC<br>CTTATAAATATTGGGTCAGGCAAAGGGATACCATTTCAGCCCAGAGTGGAGAATCGAGGGAATTTTTCCAAC<br>ACCTAGGAAATTGCTTCGGTGCGAGAAACACCTTCTGCAATCTATCAACTGCAGAAAACAGTAAAAAACATTC<br>ATCAATATTATAACATTTATATAAATACTCCAGAACTCATAGTAAATGCGCAAAGGAAGAAAAAACAATACAT<br>TCTGCAATATTTGCCAGTACCTGCCTATGTGTCATCCACTCGTGCAGCCTCCTTTCGTGCGGTGTCATCCTTCCC<br>TCCCCGGATCCTCTGGTGTAAATCCGGCCCCAACTCAAGCCTACCCATGGCACTTGTATGTTCACCACGATCAC<br>AACAGACAACCTGAGAAGAATAAGGAGGTATCGTCTAAACATTTATGGTGAAAGAAAACTCAATTTGATAAG<br>TTAAACAAAGGACGCAAGCATGACATGAGCACCAAAAACTTAAAAGATTTGCAGTTTAAGATGATTTAGAAA<br>CGAGAAGAGCGAGAGAGGAAGGAAGATAAACTTCATTTACCTACTTTCACAGTCCAATTTAGCTCTACTGCTT<br>CACTATTTTGGGCTGACATGGCGAGCCATCCCCTCGC
203	Fp-Sat203	0.024	122	0.0318	208	0.852	0.649	0.643	122	2040	GGCATTGGATGGGAACCTTGGTATGCCCACTTTTGAAGATTGTCTTGAAGAACAAGTTACCGAGAAGGGTGG<br>CTCATGGGATAATGGCCCTAACCCTAACCAGTAGATCAAGGCAGTTCTTCTTGGAATAATGTGCCTAAGTGGT<br>GCCGTCAGCGCATGTGGACTACCTTGTTGAAGACAACGCGATGGATGATATCTGCTTATCACC
219	Fp-Sat219	0.02	98	0.582	383	0.898	0.96	0.661	98	1060	GGTTTTGGATGGGTTGTTTCATCCCGATGGCATCCAGGTCCTAGACGCCCCAGCTATGGATGTCTACGATAGG<br>GTGTTGGTCTTACACTAGAGAAGATACGGTGACGAAACTGATTATAGGCATCATCTAGAAGAGGGAAGTAGT<br>TAAGAGTAACAACATGAATGAGAACAAGCTCCGATTGAGGCCGTTTAGAAAGCATTGAAATTATCTCAGGGA<br>ATTAAGATGAAATTACTTCACTTTCTCACGATCGAGATAATTTGAATGCACACACACATCCCCTCACACGTGTC<br>GAAACTCTCGACCCCCAAAACGGCTATTTAGCCACCCAAACTAACAAACAAGCTTGCAAAAAACGCATGTTAT<br>TTTTTTGTTTTTACTGTTC
236	Fp-Sat236	0.017	84	0.159	125	0.905	0.867	0.714	84	1460	AGAGGTGACGGAGAGACGAGTACAGCGTCCGCACCAAGAGGAGCACGGTACCGTGTTTCTCTTTGTTTCTCT<br>GGATGATTTCCGTTTTCTCTAGCGCAGGTACAGGTGCAAGGCTTGTGGAGTAC
